# Comparison between Intestinal Behçet's Disease and Crohn's Disease in Characteristics of Symptom, Endoscopy, and Radiology

**DOI:** 10.1155/2017/3918746

**Published:** 2017-05-31

**Authors:** Tianyu Zhang, Liwen Hong, Zhengting Wang, Rong Fan, Maochen Zhang, Yun Lin, Mengmeng Cheng, Xiaolin Zhou, Peijun Sun, Xiaoyi Lin, Jie Zhong

**Affiliations:** ^1^Department of Gastroenterology, Ruijin Hospital, Shanghai Jiao Tong University School of Medicine, Shanghai, China; ^2^Department of Laboratory Medicine, Ruijin Hospital, Shanghai Jiao Tong University School of Medicine, Shanghai, China

## Abstract

**Aim:**

To evaluate different parameters in differentiating intestinal BD from CD.

**Methods:**

The medical records of inpatients with intestinal BD and CD were retrospectively reviewed. The univariate value of different parameters was analyzed, respectively. A differentiation model was established by pooling all valuable parameters together. Diagnostic efficacy was evaluated, and a receiver operating curve (ROC) was plotted.

**Results:**

Forty-two BD patients and ninety-seven CD patients were reviewed. Demographic and clinical parameters that showed significant value included diarrhea, fever, perianal disease, oral ulcers, genital ulcers, skin lesions, and musculoskeletal lesions. Endoscopic parameters reaching clinical significance included multiple-site lesions, lesions confined to the ileocecal region, longitudinal ulcers, round or oval ulcers, punch-out ulcers, ulcers with discrete margin, ulcer size > 2 cm, stricture of bowel, and anorectal involvement. Radiologic parameters aiding the differentiation included involvement segments ≤ 3, asymmetrical pattern of involvement, intraluminal pseudopolyp formation, target sign, stricture with proximal dilation, comb sign, and fistula. The sensitivity, specificity, accuracy, positive predictive value, and negative predictive value of the differentiation model were 90.5%, 93.8%, 92.8%, 86.4%, and 95.8%, respectively. The cutoff value was 0.5 while the area under the ROC curve was 0.981.

**Conclusion:**

The differentiation model that integrated the various parameters together may yield a high diagnostic efficacy in the differential diagnosis between intestinal BD and CD.

## 1. Introduction

Behçet's disease (BD), first described by a Turkish dermatologist, is an inflammatory disorder of uncertain origin characterized by an underlying vasculitis which is highly prevalent along the Silk Road [1]. Clinical manifestations of BD include recurrent oral and genital ulcers, uveitis, and characteristic skin lesions. Also, BD patients can present with arthritis, gastrointestinal (GI) lesions, central nervous involvement, and vascular lesions [2]. BD with GI involvement is known as intestinal BD [3]. The frequency of GI involvement among BD patients varies, with lower frequency in Turkey (2.8%), India (3.4%), and Saudi Arabia (4%); moderate frequency in China (10%); and the highest frequency reported in the United Kingdom (38%–53%) and Japan (50%–60%) [4–7]. BD lesions may occur in any segment of the alimentary tract and various GI organs; however, the ileocecal region was predominantly involved.

Crohn's disease (CD) is a chronic transmural inflammatory disorder that can affect the entire GI tract from the mouth to the anus [8]. The past two decades have seen a great increase in the incidence of CD with an estimated 3-fold increase in China. Consequently, it has evolved to be one of the major health concerns of gastroenterology in China. Clinical manifestations of CD lack specificity with abdominal pain the most common followed by diarrhea, hematochezia, and weight loss [9]. An immunomodulator is a basic therapy for CD whereas surgery may be required if complications like fistula, abscess, or perforation occur [10].

Distinguishing between intestinal BD and CD is always a tough problem as they mimic each other clinically, endoscopically, and radiologically. Both the two conditions have younger age of onset, nonspecific GI symptoms, similar endoscopic features, and overlapping extraintestinal manifestations. Furthermore, lesions of both diseases could be located in any part of the alimentary tract, with the ileocecal region involved mostly [11–12]. Moreover, both these two disorders have a waxing and waning disease course while immunomodulators may be therapeutically effective. In an era of precision medicine, it is imperative to differentiate intestinal BD from CD [13–14]. For similar but not the same therapeutic strategy, a precise diagnosis and a thorough evaluation may bring benefit to treatment, thus improving the prognosis of the disease. In this study, we retrospectively reviewed the clinical manifestations, endoscopic features, and radiological characteristics of intestinal BD and CD in our hospital and tried to develop a potential model for differentiating these two conditions.

## 2. Methods

### 2.1. Patients Enrolled

A retrospective study of a single medical center was designed. The medical records of inpatients with intestinal BD and CD treated from February 1, 2004, to September 15, 2016, in Ruijin Hospital, Shanghai, China, were reviewed. All enrolled patients had undergone endoscopy as well as CT enterography (CTE) or MR enterography (MRE) in our hospital. Moreover, at least one-year follow-up after diagnosis was required for these patients. All enrolled patients had undergone T-SPOT (−) to exclude existing TB infection.

### 2.2. Diagnostic Criteria

All enrolled BD patients had gastrointestinal involvement. A diagnosis of BD was based on the criteria suggested by the BD Research Committee of Japan in 1987 [15]. Four major features included recurrent oral aphthous ulcers, ocular lesions, genital aphthous ulcers, and cutaneous hypersensitivity. Minor features of BD were comprised of arthritis, gastrointestinal lesions, epididymitis, vascular lesions, and central nervous system involvement. All BD patients had been referred to the rheumatologists who were specialized in BD for consultation and evaluation to confirm each typical lesion. There were three types of BD: (1) complete type—meeting four major features; (2) incomplete type—meeting three major features, or two major and two minor features, or typical ocular lesions plus another major or two minor features; and (3) suspected type—meeting two major features or one major and two minor features. In our study, the complete, incomplete, and suspected types of BD were 7 (16.7%), 22 (52.4%), and 13 (31.0%), respectively. 38/42 (90.5%) patients fulfilled the diagnostic criteria of the International Study Group for BD (ISGBD) [16], which did not include gastrointestinal lesions.

A diagnosis of CD was based on morphological (radiological, endoscopic, or surgical findings) and pathological criteria suggesting focal, asymmetrical, transmural, or granulomatous features [17]: (1) morphological—(a) discontinuous/segmental and asymmetrical mucosal involvement, (b) deep mucosal longitudinal fissures/ulcers, (c) transmural inflammation, (d) rigid and strictured intestinal wall, and (e) presence of enterocutaneous/enteroenteric fistula and/or chronic perianal disease and/or other extraintestinal complications and (2) pathological—(a) normal mucus content in the goblet cells of the inflamed region, (b) lymphocyte aggregation in the mucosa and submucosa, (c) noncaseating granuloma, (d) longitudinal ulcers/fissures, and (e) transmural inflammation or inflammation beyond the mucosa. Diagnosis of CD should meet the following criteria: presence of at least 3 different criteria or presence of noncaseating granuloma on histology with at least 1 other criterion.

### 2.3. Clinical Evaluation and Data Collected

Demographic, clinical, endoscopic, and radiologic data were collected at the time of initial diagnosis when the patients were naïve to immunomodulators' therapy.

The demographic data included patients' sex, age of onset, and BMI. Clinical manifestations were duration of symptoms, abdominal pain, diarrhea, abdominal distension, nausea, hematochezia, abdominal mass, fever, and weight loss. Extraintestinal manifestations included perianal disease, oral ulcers, genital ulcers, skin lesions, ocular lesions, musculoskeletal lesions, vascular abnormality, and neurologic lesions.

Double-balloon enteroscopy (DBE) was performed if the lesion was confined to the small intestine, while colonoscopy was performed for patients with only colonic involvement. Endoscopic features included multiple-site lesions, lesions confined to the ileocecal region, deformity of ileocecal valve, longitudinal ulcers, round and oval ulcers, punch-out ulcers, ulcers with discrete margin, ulcer size > 2 cm, cobblestone appearance, aphthous lesions, stricture of bowel, and anorectal involvement.

All of our enrolled patients had undergone CTE or MRE at least once which was evaluated by an experienced radiologist independently. Radiologic characteristics mainly included involvement of ≤3 segments, thickening bowel wall, asymmetrical pattern of involvement, intraluminal pseudopolyp formation, target sign, circumintestinal exudation, stricture with proximal dilation, comb sign, fistula, abscess, phlegmon, and ascites.

### 2.4. Statistical Analyses

SPSS 19.0 was used for the data analyses and screening for potential valuable parameters for differential diagnosis between CD and BD. Continuous variables were expressed as mean ± SD, and a comparison was performed by using the Student *t*-test if the data had a normal distribution. Median values (upper and lower quartiles) were calculated, and the Wilcoxon rank sum test was used to analyze the data that did not have a normal distribution. Binary categorical variables were expressed as frequency and percentage values, while comparisons were made using the chi-square or Fisher's exact test. A probability (*P*) value of <0.05 was considered to be statistically significant. Then, continuous variables were converted to binary categorical variables based on the Youden index. All of the parameters with significant differences in differentiating diagnosis were graded, with 1 in CD and −1 in BD. A differentiation model was created by adding all of the scores of the valuable parameters. The total score was calculated, and a receiver operating characteristic (ROC) curve was plotted. The cutoff value was obtained from the Youden index. Sensitivity, specificity, accuracy, PPV, and NPV were calculated to evaluate the diagnostic efficacy of the model.

## 3. Results

### 3.1. Demographic Characteristics and Clinical Manifestations of Intestinal BD and CD

The medical records of 42 BD patients and 97 CD patients were reviewed. The demographic characteristics and clinical manifestations of intestinal BD and CD are summarized in Table 1. No significant difference was found in terms of patients' sex, age of onset, and BMI. The duration of symptoms was quite similar between intestinal BD and CD. For clinical manifestations, the occurrence of diarrhea and perianal disease was more often seen in CD than in intestinal BD (*P* < 0.05). In contrast, the occurrence of fever and extraintestinal symptoms including oral ulcers, genital ulcers, skin lesions, and musculoskeletal lesions in intestinal BD was significantly higher than that in CD (*P* < 0.05). For other clinical manifestations, including abdominal pain, abdominal distension, nausea, hematochezia, abdominal mass, weight loss, ocular lesions, vascular abnormality, and neurologic lesions, no significant difference was found between these two conditions.

### 3.2. Endoscopic Features of Intestinal BD and CD

**Figure 1:**
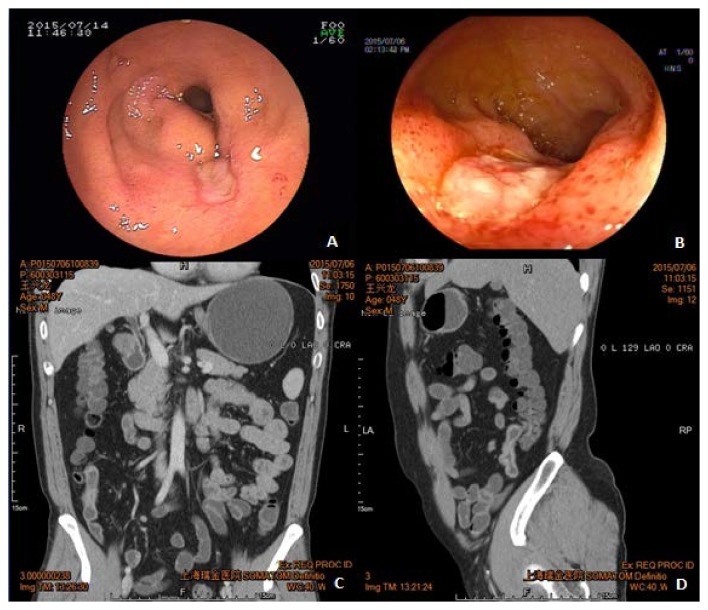
Endoscopic and CTE features in intestinal BD. (a, b) Colonoscopy revealed oval punch-out ulcer with discrete margin in the ileocecal region. (c, d) CTE showed the same patient with thickening bowel wall and circumintestinal exudation in the terminal ileum.

**Figure 2:**
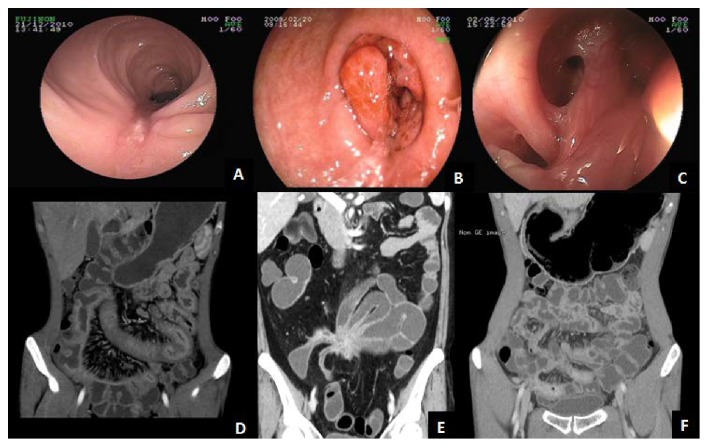
Endoscopic and CTE features in CD: (a) longitudinal ulcer that stretched across several folds under endoscopy; (b) bowel obstruction in the ascending colon due to chronic inflammation; (c) perianal fistula with multiple internal openings; (d) stretching and densifying of distal mesenteric artery the so-called comb sign in the ileum; (e) internal bowel-bowel fistula the so-called petal sign; (f) asymmetrical thickening of the bowel wall with target sign.

The endoscopic features of intestinal BD and CD are listed in Table 2. Lesions in CD patients tended to involve multiple sites compared with those in intestinal BD (*P* < 0.05). In contrast, compared with those of CD, lesions of intestinal BD were more confined to the ileocecal region (*P* < 0.05). The morphology of ulcers under endoscopy differed between intestinal BD and CD. Round or oval ulcers and punch-out ulcers (Figures 1(a) and 1(b)) were more common in intestinal BD (*P* < 0.05), whereas longitudinal ulcers (Figure 2(a)) were more apparent in CD (*P* < 0.05). Moreover, intestinal BD patients were more likely to have ulcers > 2 cm and with discrete margin (Figures 1(a) and 1(b)) than CD patients (*P* < 0.05). On the contrary, cobblestone appearance, stricture of bowel (Figure 2(b)), and anorectal involvement (Figure 2(c)) were more frequently found in CD patients than in intestinal BD patients (*P* < 0.05). For other parameters including deformity of ileocecal valve and aphthous lesions, no significant difference was found between these two groups.

### 3.3. Radiologic Findings of Intestinal BD and CD

For radiologic findings shown in Table 3, only involvement segments ≤ 3 indicated a diagnosis of intestinal BD instead of CD (*P* < 0.05). Compared with intestinal BD patients, CD patients had characteristic lesions under CTE or MRE including asymmetrical pattern of involvement, intraluminal pseudopolyp formation, and target sign (Figure 2(f)) (*P* < 0.05). Moreover, stricture with proximal dilation was significantly higher in CD than in intestinal BD. For extraintestinal manifestation, the occurrence of comb sign (Figure 2(d)) and fistula (Figure 2(e)) favored a diagnosis of CD compared with that of intestinal BD. No significant difference was found between these two groups in terms of thickening bowel wall, circumintestinal exudation (Figures 1(c) and 1(d)), abscess, phlegmon, and ascites.

### 3.4. Total Score of the Differential Parameters, Diagnostic Efficacy of the Differentiation Model, and ROC Curve

**Figure 3:**
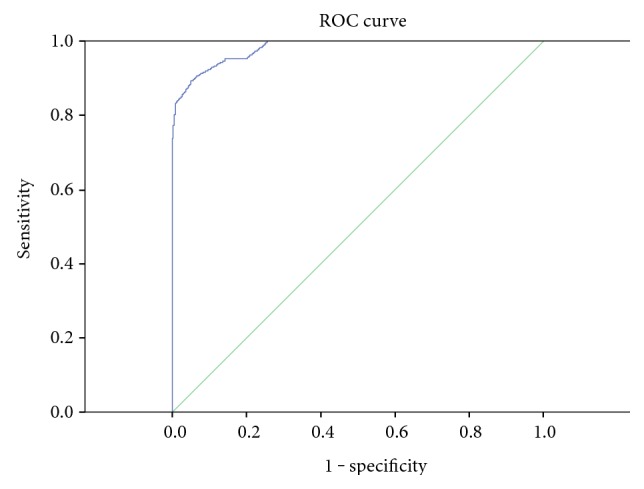
ROC curve of the differentiation model (area under the ROC curve is 0.981).

The total score was calculated by pooling all of the valuable differential parameters together. A differentiating diagnostic model was established, with high sensitivity (90.5%), specificity (93.8%), accuracy (92.8%), PPV (86.4%), and NPV (95.8%). A ROC curve was plotted. Based on the Youden index, a diagnostic point of 0.5 was obtained (*P* > 0.5, predictable diagnosis of intestinal BD; *P* < 0.5, diagnosis of CD), and the area under the ROC curve was 0.981 (Figure 3).

## 4. Discussion

BD is a systematic disease mainly characterized by oral, genital, ocular, and skin lesions. Sometimes, BD patients can present with GI ulcers, the so-called intestinal BD. CD is a chronic inflammatory GI disease which may also have extraintestinal manifestations mimicking BD greatly [18–20]. Moreover, morphology of GI ulcers between the two conditions is quite similar, making differential diagnosis a tough problem for clinicians [12]. There have been several studies focusing on the differential diagnosis between intestinal BD and CD in recent years [11, 12, 18, 21]. But to the best of our knowledge, no diagnostic algorithm has been established collating numerous parameters together. In this study, we retrospectively reviewed the demographic, clinical, endoscopic, and radiologic parameters of inpatients with intestinal BD and CD. After screening out parameters with clinical significance, we used them to establish a differentiation model that is more objective and more convenient thus boosting the differential diagnosis between intestinal BD and CD.

Among various demographic and clinical parameters, our study showed that diarrhea, fever, and perianal disease were most useful in differentiating intestinal BD from CD. Among them, fever favored a diagnosis of intestinal BD, whereas diarrhea and perianal disease favored a diagnosis of CD. These had further proved that intestinal BD and CD had overlapping clinical manifestations. For several systematic symptoms listed in the diagnostic criteria of BD, we found that only four of them, including oral, genital, skin, and musculoskeletal lesions, aided the differential diagnosis. Thus, it could be inferred that systematic symptoms of BD could mimic extraintestinal manifestations of CD. Consequently, we could not make a differential diagnosis between the two conditions only according to the symptoms. Our findings were similar to those of Li et al. [21].

Endoscopy is the first choice for clinicians to detect bowel lesions and evaluate therapeutic response. In our study, we found that the ulcers' distribution of the two diseases was different. CD patients tended to have multiple-site involvement, whereas lesions of intestinal BD were more likely to be confined to the ileocecal region. Moreover, the morphology of the lesions was different from each other. Ulcers of intestinal BD were always round or oval in shape, punch-out in feature, >2 cm in size, and with a discrete margin. On the other hand, ulcers of CD were mostly longitudinal in shape. Cobblestone appearance, stricture of bowel, and anorectal involvement in CD patients may help distinguishing them from patients with intestinal BD. Our study has proved that although quite similar, some distinctions do exist between intestinal BD and CD in endoscopy, which is helpful to the differential diagnosis. These findings are in good agreement with those of Lee et al. [12].

CTE and MRE are emerging noninvasive technology for the diagnosis and evaluation of small-bowel diseases. Compared with endoscopy, they have a better diagnostic efficacy of both bowel wall lesions and extraenteric manifestations [22]. Moreover, they act as good tools in the differentiation of CD from other diseases which have been proved by our former researches [23, 24]. Our study illustrated that only involvement segments ≤ 3 favored a diagnosis of intestinal BD compared with that of CD. Although other parameters including thickening bowel wall and circumintestinal exudation had higher sensitivity in intestinal BD, they served as a poor value in differential diagnosis. These had further proved that radiologic findings of intestinal BD lack specificity. On the contrary, the occurrence of asymmetrical pattern of involvement, intraluminal pseudopolyp formation, target sign, stricture with proximal dilation, comb sign, and fistula was significantly higher in the CD group compared with the intestinal BD group. Consequently, it can be inferred that radiologic findings had high specificity in CD, which were very helpful in differentiating these two diseases.

Although a series of differentiating parameters had been proposed, none of them enjoyed high sensitivity and specificity at the same time. As a result, differentiating between these two conditions through a single parameter is really difficult. Thus, we graded each parameter and established a diagnostic model that combined all of the valuable parameters together. Through later statistical analysis, we proved that our model had high diagnostic efficacy, with high sensitivity (90.5%), specificity (93.8%), accuracy (92.8%), PPV (86.4%), and NPV (95.8%). Based on the Youden index, we acquired a cutoff value of 0.5 and the area under the curve was 0.981. We believe that our differentiation model is more integrated and serve better, helping clinical practitioners to solve this problem.

This study has some limitations. First, as its retrospective nature and limited number of patients, the level of evidence is limited. We expect more prospective studies and multicenter collaboration being carried out regarding this field. Second, we did not include other ulcerous bowel disease into the analysis. Thus, this differentiating model could be used only if other diseases had been excluded, which may hamper its application. Third, all of the enrolled patients were inpatients in our hospital, which may bring selection bias.

In conclusion, intestinal BD and CD have overlapping characteristics making it hard to distinguish from each other. However, some parameters including clinical manifestations, endoscopic features, and radiologic characteristics are valuable in differentiating these two conditions. The established differentiating model that collated different parameters together yields high diagnostic efficacy, which will be very helpful in clinical practice.

## Figures and Tables

**Table 1:** Demographic characteristics and clinical manifestations of intestinal BD and CD.

Features	Intestinal BD (*N* = 42)	CD (*N* = 97)	*P* value	Score
Sex (male/female)	20/22	54/43	0.382	N/A
Age of onset	39.3 ± 17.3	36.9 ± 16.4	0.384	N/A
BMI (kg/m^2^)	21.63 ± 3.2	21.08 ± 4.3	0.394	N/A
Duration of symptoms (months)	25.2 ± 23.8	25.0 ± 24.3	0.969	N/A
Abdominal pain	27 (64.3)	75 (77.3)	0.110	N/A
Diarrhea	13 (31.0)	65 (67.0)	<0.001	−1
Abdominal distension	11 (26.2)	32 (33.0)	0.426	N/A
Nausea	4 (9.5)	14 (14.4)	0.429	N/A
Hematochezia	11 (26.2)	25 (25.8)	0.959	N/A
Abdominal mass	2 (4.8)	6 (6.2)	1.000	N/A
Fever	14 (33.3)	14 (14.4)	0.011	1
Weight loss	16 (38.1)	51 (52.6)	0.117	N/A
Perianal disease	5 (11.9)	42 (43.3)	<0.001	−1
Oral ulcers	42 (100.0)	32 (33.0)	<0.001	1
Genital ulcers	29 (69.0)	14 (14.4)	<0.001	1
Skin lesions	23 (54.8)	8 (8.2)	<0.001	1
Ocular lesions	3 (7.1)	2 (2.1)	0.326	N/A
Musculoskeletal lesions	15 (35.7)	8 (8.2)	<0.001	1
Vascular abnormality	5 (11.9)	7 (7.2)	0.565	N/A
Neurologic lesions	2 (4.8)	1 (1.0)	0.217	N/A

**Table 2:** Endoscopic features of intestinal BD and CD.

Features	Intestinal BD (*N* = 42)	CD (*N* = 97)	*P* value	Score
Multiple-site lesions	22 (52.4)	87 (89.7)	<0.001	−1
Lesions confined to the ileocecal region	38 (90.5)	20 (20.6)	<0.001	1
Deformity of ileocecal valve	8 (19.0)	19 (19.6)	0.941	N/A
Longitudinal ulcers	5 (11.9)	85 (87.6)	<0.001	−1
Round or oval ulcers	33 (78.6)	29 (29.9)	<0.001	1
Punch-out ulcers	25 (59.6)	13 (13.4)	<0.001	1
Ulcers with discrete margin	33 (78.6)	59 (60.8)	0.042	1
Ulcer size > 2 cm	22 (52.4)	32 (33.0)	0.031	1
Cobblestone appearance	5 (11.9)	31 (32.0)	0.013	−1
Aphthous lesions	22 (52.4)	45 (46.4)	0.516	N/A
Stricture of bowel	3 (7.1)	23 (23.7)	0.021	−1
Anorectal involvement	3 (7.1)	21 (21.6)	0.038	−1

**Table 3:** Radiologic findings of intestinal BD and CD.

Features	Intestinal BD (*N* = 42)	CD (*N* = 97)	*P* value	Score
Involvement segments ≤ 3	32 (76.2)	37 (38.1)	<0.001	1
Thickening bowel wall	37 (88.1)	89 (91.8)	0.717	N/A
Asymmetrical pattern of involvement	6 (14.3)	40 (41.2)	<0.001	−1
Intraluminal pseudopolyp formation	8 (19.0)	58 (59.8)	<0.001	−1
Target sign	2 (4.8)	21 (21.6)	<0.001	−1
Circumintestinal exudation	31 (73.8)	74 (76.3)	0.755	N/A
Stricture with proximal dilation	4 (9.5)	24 (24.7)	0.040	−1
Comb sign	7 (16.7)	73 (75.3)	<0.001	−1
Fistula	1 (2.4)	37 (38.1)	<0.001	−1
Abscess	3 (7.1)	12 (12.3)	0.539	N/A
Phlegmon	1 (2.4)	8 (8.2)	0.360	N/A
Ascites	0 (0.0)	6 (6.2)	0.233	N/A
